# Investigating Topical Steroid Withdrawal Videos on TikTok: Cross-Sectional Analysis of the Top 100 Videos

**DOI:** 10.2196/48389

**Published:** 2024-08-29

**Authors:** Firas Haddad, William Abou Shahla, Dana Saade

**Affiliations:** 1 Faculty of Medicine American University of Beirut Medical Center Beirut Lebanon; 2 Department of Dermatology American University of Beirut Medical Center Beirut Lebanon

**Keywords:** steroid withdrawal, medical dermatology, drug response, social media, videos, TikTok, steroids, content analysis, information quality, skin, topical, dermatology, misinformation

## Abstract

**Background:**

Social media platforms like TikTok are a very popular source of information, especially for skin diseases. Topical steroid withdrawal (TSW) is a condition that is yet to be fully defined and understood. This did not stop the hashtag #topicalsteroidwithdrawal from amassing more than 600 million views on TikTok. It is of utmost importance to assess the quality and content of TikTok videos on TSW to prevent the spread of misinformation.

**Objective:**

This study aims to assess the quality and content of the top 100 videos dedicated to the topic of TSW on TikTok.

**Methods:**

This observational study assesses the content and quality of the top 100 videos about TSW on TikTok. A total of 3 independent scoring systems: DISCERN, *Journal of the American Medical Association*, and Global Quality Scale were used to assess the video quality. The content of the videos was coded by 2 reviewers and analyzed for recurrent themes and topics.

**Results:**

This study found that only 10.0% (n=10) of the videos clearly defined what TSW is. Videos were predominantly posted by White, middle-aged, and female creators. Neither cause nor mechanism of the disease were described in the videos. The symptoms suggested itching, peeling, and dryness which resembled the symptoms of atopic dermatitis. The videos fail to mention important information regarding the use of steroids such as the reason it was initially prescribed, the name of the drug, concentration, mechanism of usage, and method of discontinuation. Management techniques varied from hydration methods approved for treatment of atopic dermatitis to treatment options without scientific evidence. Overall, the videos had immense reach with over 200 million views, 45 million likes, 90,000 comments, and 100,000 shares. Video quality was poor with an average DISCERN score of 1.63 (SD 0.56)/5. Video length, total view count, and views/day were all associated with increased quality, indicating that patients were interacting more with higher quality videos. However, videos were created exclusively by personal accounts, highlighting the absence of dermatologists on the platform to discuss this topic.

**Conclusions:**

The videos posted on TikTok are of low quality and lack pertinent information. The content is varied and not consistent. Health care professionals, including dermatologists and residents in the field, need to be more active on the topic, to spread proper information and prevent an increase in steroid phobia. Health care professionals are encouraged to ride the wave and produce high-quality videos discussing what is known about TSW to avoid the spread of misinformation.

## Introduction

### Background

Social media is rapidly integrating into the health care domain and becoming an essential part of health care delivery. More and more health care workers are resorting to social media to spread awareness and debunk false information, and more people are resorting to social media to acquire medically related information [1]. TikTok, in particular, is the fastest-growing platform with over 1 billion active users per month [2,3]. TikTok allows users to view videos specifically curated for them and their personal interests through a feature called “For You Page.” This allows both video producers to reach higher number of people and for people to access millions of videos they are interested in through a simple interface [2]. As such, health care–related TikTok videos have a very wide reach and high rates of interactions [4].

Topical steroids are frequently used in the field of dermatology as the first line of treatment for many skin conditions such as atopic dermatitis, eczema, psoriasis, and others [5]. Although there are specific protocols for the indications, and proper usage of steroids, some patients misuse this class of medications [6]. A minuscule fraction of 5.5% of patients possess knowledge regarding the specific medication used and the potential side effects involved, with only a mere one-third demonstrating an understanding of the appropriate indications for this treatment [7]. The improper use and abrupt discontinuation of the medication may result in adverse effects, including rarely topical steroid withdrawal [8]. It is important to distinguish the particular topical steroid used and the extent of the body surface area affected when looking at the topical steroid withdrawal syndrome. It is equally plausible as well, that the underlying disease itself is accountable for the emergence of new atopic conditions, rather than the use of topical steroids [9]. Patients who have experienced such side effects are resorting to social media to share their experiences under the syndrome of “topical steroid withdrawal (TSW),” a hashtag that has accumulated 622.7 million views on TikTok, although it is yet to be properly defined as a discrete condition [10].

### Objective

Given the fact that TSW videos have such a wide reach on TikTok, the videos’ credibility and accuracy need to be evaluated and scrutinized [11]. This study aims to analyze the top 100 videos on the topic of TSW on TikTok, possible recurrent themes or definitions of this condition, and the video quality.

## Methods

### Study Design

The hashtag #topicalsteroidwithdrawal was queried on the TikTok search page. The first 100 videos on the “Top Videos” page of the search were included in the study. The videos were independently assessed by 2 reviewers. The first 5 videos were assessed by the 2 reviewers (FH and WAS) to calibrate the data extraction process, and any differences were sorted out by consulting a third reviewer (DS), who was blind to the extraction of the first 2 reviewers. Data extraction was based on what was being said in the videos and not on assumptions, visual signs, and other videos posted by the account, and all information was coded onto Microsoft Excel. As for the patients’ age group, the latter was categorized into infant, middle-aged, or elderly based on visual information in the videos

The videos were extracted on January 15, 2023. The time on TikTok was assessed as the date of the publication of the video till January 15, 2023. The number of daily views was calculated as total views/time on TikTok.

### Video Quality

Video quality was assessed by 3 scoring scales. The first is the DISCERN scoring instrument [12], which consists of 15 questions each getting a score between 1 and 5. The total score of the 15 questions is then divided by 15 to yield an average score ranging from 1 to 5. The second tool is the *Journal of the American Medical Association* (*JAMA*) scoring system [13], which consists of 4 questions each getting a point of either 0 or 1. The last score is the Global Quality Scale (GQS) score [13], which is a 1-choice assessment ranging from 1 to 5. Higher points on all 3 scales indicate higher quality. Mean scores were computed. These tools have been widely used in studies to assess the quality of health care–related content on the internet [13,14].

### Data Analysis

Data analysis was done on Microsoft Excel and Python Anaconda (Anaconda, Inc). Means, SDs, and ranges were computed in Excel. Correlations were computed on Python Anaconda.

### Ethical Considerations

TikTok’s data and video content are available to the public, and thus, this study is exempt from institutional board review. Data used and shared does not contain any identifiers for the video creators.

## Results

### Content Analysis

#### Definition, Demographics, Causes, and Mechanism of Disease

A total of 100 videos were included in the study. Of those, only 10 videos gave a clear definition of TSW with 4 out of them defining it as an addiction to topical steroids, and the loss of skin tolerance to it, which culminates in the development of the condition.

Table 1 highlights the main demographics, suggested causes and mechanisms of TSW, signs, and symptoms.

Table 2 outlines the number of videos that did and did not mention information related to the data parameters extracted.

**Table 1:** Main characteristics of TSW^a^ including demographics, suggested causes and mechanism of TSW, symptoms, and signs.

Characteristics						Videos (N=100), n (%)
**Demographics (N=100)**						
	**Sex**					
		Female			71 (71.0)	
		Male			29 (29.0)	
	**Age**					
		Middle-aged			100 (100.0)	
	**Ethnicity**					
		Asian			19 (19.0)	
		Black			13 (13.0)	
		White			68 (68.0)	
**Medical Explanation for TSW according to the videos (n=17)**						
	Mention “eczema”					7 (7.0)
	Reaction to stopping the steroid					5 (5.0)
	Overprescription and overuse of steroids without proper patient education					4 (4.0)
	Skin reaction to gastrointestinal dysregulation					1 (1.0)
**Suggested mechanism of TSW (n=9)**						
	Exogenous steroids cause negative feedback on the adrenal gland that halts its production of cortisol, making the skin dependent on exogenous steroids					7 (7.0)
	Destruction of gut after overconsumption of antibiotics					1 (1.0)
	Highly dependent on the environment					1 (1.0)
**Symptoms (n=60)**						
	**Short-term symptoms**					
		Itchiness				22 (22.0)
		Peeling, shedding, and flaking				19 (19.0)
		Dryness and dehydration				19 (19.0)
		Pain				13 (13.0)
		Redness, burning, and red skin syndrome				15 (15.0)
		Oozing and pus				10 (10.0)
		Limitation of movement				7 (7.0)
		Tiredness				5 (5.0)
		Mental health issue				5 (5.0)
		Bleeding				2 (2.0)
		Sweating				2 (2.0)
		Hair loss				2 (2.0)
		Insomnia				4 (4.0)
		Scaling and crusting skin				3 (3.0)
		Irritated skin and allergy to food				1 (1.0)
		Elephant skin				1 (1.0)
		Spider veins				1 (1.0)
	**Long-term complication**					
		Inability to go to work				10 (10.0)
		Mental health issues such as unspecific mental problems, panic attack, PTSD^b^, suicidal attempt				7 (7.0)

^a^TSW: topical steroid withdrawal.

^b^PTSD: posttraumatic stress disorder.

**Table 2:** Main data extracted from the 100 videos on TikTok (N=100)^a^.

	Not mentioned, %	Mentioned, %
Mention the condition as “TSW^b^”	81.0	19.0
Definition of TSW	90.0	10.0
Cause of disease	83.0	17.0
TSW as a disability	96.0	4.0
Mechanism of disease	91.0	9.0
Potency of steroid used	93.0	7.0
Duration of use	86.0	14.0
Reason of stopping	89.0	11.0
Method of discontinuation	95.0	5.0
Duration since discontinuation	90.0	10.0
Symptoms experienced	40.0	60.0
Time of onset of symptoms	89.0	11.0
Inability to go to work	90.0	10.0
Mention TSW as a disability	96.0	4.0
Duration of withdrawal symptoms	75.0	25.0
Management	51.0	49.0

^a^Since the video count was N=100, the number and percent are identical.

^b^TSW: topical steroid withdrawal.

#### Steroids Used and Reasons for Discontinuation

Only 7.0% (n=7) reported the type of corticosteroids used prior to their discontinuation. Steroids prescribed include the combination hydrocortisone-miconazole, betamethasone, mometasone, and oral prednisone. The data regarding the duration of usage was mentioned by a minority of the content creators (14.0%, n=14), and it ranged from 8 months to a few years.

The reason for stopping the steroids was not mentioned in 90.0% (n=90) of the videos. The remaining (10.0%, n=10) gave the following justifications: aggravation of the condition (4.0%, n=4), ineffectiveness of TCS (2.0%, n=2), steroid phobia (1.0%, n=1), pregnancy (1.0%, n=1), change of environment (1.0%, n=1), personal decision (1.0%, n=1), and being advised by a friend (1.0%, n=1). Around 5.0% (n=5) of the videos explicitly mentioned the abrupt discontinuation of the steroids.

#### Symptoms

The symptoms of TSW were mentioned in 60 videos only, which we divided into short-term symptoms, and long-term complications, noting the major signs and symptoms. Table 2 describes the signs and symptoms in more detail.

These symptoms started either directly after stopping steroids (4.0%) or 6 to 18 months after discontinuation (7.0%). A total of 25 videos mentioned the duration of TSW, and it ranged between 3 months reaching up to 3 years.

#### Management

Table 3 outlines the main management techniques mentioned in the different videos.

**Table 3:** Management tools for TSW^a^ and its complication according to the content creators^b^.

Management			Values (n=49), n
**Alone by myself**			
	Dietary products, natural products		7
	Body thermoregulation (bath, fan, and facials)		6
	**Topical cream**		
		Self-made (tea tree oil and manuka honey)	2
		Petroleum jelly	3
**Someone’s help**			
	Family and husband emotional support		2
	Wrapped ice packs		4
	Daily moisturizer		1
**Doctor’s suggestion**			
	Use steroid again (gradually increase the dose)		3
	Upadacitinib		2
	Betamethasone		1
	Ointments and moisturizers		3
	Immunosuppressants (Dupilumab, Methotrexate)		1
	**Other medications**		
		Antibacterial creams	1
		Tacrolimus	1
**Others**			
	No moisture treatment (avoid topicals, water intake, and showers)		12
	Chinese medicine		5
	Sunbeds		2
	Goes away completely with time, no treatment		4
	Stop using makeup, soap, and alcohol		5
	Use arthritis gloves		2
	Alcohol wipes		1
	Meditation		1

^a^TSW: topical steroid withdrawal.

^b^Since the video count was N=100, the number and percent are identical.

### Quality Analysis

#### Reach of Videos

The study included 100 videos, with a total duration of 6158 seconds, and the total view count of these 100 videos included is 201,722,910 views, 48,970,260 likes, 93,276 comments, 120,065 shares, and 321,508 saves. The earliest video published was on September 15, 2020, and the latest video was published on December 26, 2022. All the videos included in this study were published by personal accounts, and none were published by health care professionals, brands, and organizations. Table 4 includes the mean, SD, and range of different data parameters.

**Table 4:** Mean (SD) and the range of the video parameters.

	Mean (SD)	Range
Video length (in seconds)	61.58 (55.97)	5-218
Days on TikTok	269.69 (138.09)	56-711
Number of views	2017229.10 (4825328.89)	20,300-38,500,000
Number of daily views	10141.27 (17934.30)	76.44-76056.34
Number of likes	489702.60 (3407573.30)	153-33,900,000
Number of comments	961.61 (1813.10)	0-9823
Number of shares	1200.65 (3244.26)	0-26,100
Number of saves	3215.08 (12695.86)	2-118,000
DISCERN	1.63 (0.56)	1-3.46
*JAMA^a^*	1.30 (0.56)	1-3
GQS^b^	1.71 (0.84)	1-4

^a^JAMA: Journal of the American Medical Association.

^b^GQS: Global Quality Score.

#### Scoring

The mean DISCERN score was 1.63 (SD 0.56, range 1-3.46), the mean *JAMA* score was 1.30 (SD 0.56, range 1-3), and the mean GQS was 1.71 (SD 0.85, range 1-4). Table 5 includes the correlation between the different scores and different data parameters. Data shows that the video length, daily views, and total views were positively correlated with all 3 scoring systems. Higher quality videos across all 3 scoring systems were correlated with increased total and daily view count, indicating that higher quality videos were more likely to be viewed and interacted with than their lower quality counterpart.

**Table 5:** Correlations among the 3 scoring systems and different video parameters.

	DISCERN		*JAMA^a^*		GQS^b^	
	Mean (SD)	*P* value	Mean (SD)	*P* value	Mean (SD)	*P* value
DISCERN	1	N/A^c^	0.55	N/A	0.74	N/A
*JAMA*	0.55	N/A	1	N/A	0.61	N/A
GQS	0.74	N/A	0.61	N/A	1	N/A
Video length	0.47	.001	0.24	.002	0.53	<.001
Total views	0.32	.001	0.47	<.001	0.32	<.001
Views/day	0.244	.01	0.48	<.001	0.28	.004
#Likes	–0.053	.60	–0.032	.75	–0.068	.50
#Comments	0.089	.38	0.11	.28	0.13	.19
#Shares	0.13	.20	0.17	.01	0.16	.11
#Saves	0.068	.50	0.20	.40	0.16	.12

^a^*JAMA*: *Journal of the American Medical Association*.

^b^GQS: Global Quality Score.

^c^N/A: data are not applicable or not available.

## Discussion

### Principal Findings

Regarding the video content, the information shared in the videos lacked several key information about the TSW syndrome but also contained insightful information for practitioners. One of the major shortcomings in these TikTok videos is the lack of information on their initial condition, only 18 videos disclosed that, 7 mention the steroid used, 14 the duration of steroid use, 11 the reason for discontinuation, and 5 the method of discontinuation. The duration of steroid use was missing in 86% of the videos, and when mentioned, it ranged between a few months to several years, and as such would be considered long-term steroid users. These are important pieces of information to elicit as only long-term use of moderate and high-potency corticosteroids has been associated with the development of side effects like the ones presented in the videos [10,15]. The limited data from the included videos suggests that the steroids may have been improperly used, discontinued without the agreement of a dermatologist, or discontinued in an improper manner.

From the videos, 89% did not mention the reason for discontinuation, and the videos that did, say that it was due to worsening of symptoms, development of adverse effects, and moving to new environments [16]. Five of the videos mentioned abrupt discontinuation of the steroids, and none mentioned tapering them off. Tapering steroids off is an important factor in avoiding a flare of the initial dermatological conditions, and the prevention of withdrawal symptoms to occur [17]. The appropriate step-down and step-up approaches can help mitigate these withdrawal symptoms and help the patient to cope well with the condition [18].

Four of the videos mentioned that the TSW symptoms occurred directly after stopping steroids, and others mentioned the appearance of symptoms months after. However, in a study carried out by Sheary et al [19], TSW occurs after 4-6 weeks of TCS discontinuation, which raises the question of whether the symptoms mentioned in the videos are due to a distinct entity of TSW or a flare-up of the initial condition.

Another limitation of the videos is the vague definition of the condition. Only 10% of the videos mentioned a clear definition of what TSW is, and of that 10%, some defined it as being simply a condition separate from eczema, without disclosing more details on this condition. Two of the content creators mentioned that the use of exogenous steroids suppressed the adrenal gland’s own production of steroids, making the body dependent on exogenous steroids. This is the rationale behind the gradual discontinuation of corticosteroids [20]. Rapid and abrupt discontinuation of steroids does not allow the adrenal gland to regain its function and thus generates the side effects of steroid use [21]. Conversely, another study showed that abrupt discontinuation of steroids may lead to the reappearance of the initial condition [10,19,22,23]. Considering all that mentioned previously, we cannot ascertain as to whether the symptoms experienced in these videos are a flare of their initial condition, a new entity altogether, or indeed topical steroid withdrawal syndrome.

A few of the side effects reported in the literature after misusing TCS were facial acne, telangiectasia, cutaneous atrophy, and facial or perioral dermatitis [24] In this study, the most described symptoms were itchiness and a rash that affected the whole body including the face. Itchiness and rash are very broad and nonspecific and do not indicate a particular condition, withdrawal or not.

The red skin syndrome denoting generalized redness was mentioned in the videos. Based on reported studies on this topic, red skin syndrome could be a potential diagnostic criterion for TSW [19,25]. Elephant skin which was reported in 2 cases is described as thickened skin with a reduction in skin elasticity. Although its association with TSW was newly described in the literature, it has been considered 1 possible manifestation of steroid misuse [19]. Long-term complications of TSW included mental disturbances in several videos. A study highlights how these same mental problems are reported in cases of long-term usage of steroids, and thus are not necessarily a manifestation of withdrawal [16].

No consistent management technique was found among video creators. Dietary modifications, homemade creams, and Chinese medicine were all mentioned in several instances. All 3 of these management techniques were described for dermatitis, and for TSW, and they lacked high-quality evidence [26,27]. “no moisture treatment” where one restricts all forms of hydration was mentioned several times and goes against the scientifically-backed evidence that moisturizers and hydration are essential to maintain or restore skin barrier.

Our study highlights both the high reach of the TSW videos on TikTok, as well as the poor quality of the videos which may contribute to the spreading of misinformation on steroid use. The videos have been viewed a total of 201.7 million times, which indicates a huge reach, and that users are actively watching and searching for content on topical steroid withdrawal. Users are also interacting with those videos by liking, commenting, sharing, and saving. The huge reach of these videos shows that TikTok may serve as a gateway of information for its users on topical steroid withdrawal. A study by Marar et al [28] showed that 85% use social media for health information. TikTok is emerging as a popular source for acquiring health information, and that is proven by the huge reach that TSW videos have on TikTok.

With that being said, it is important that these videos be of good quality in order not to spread false information. Our study uses 3 different tools to assess the quality of health care videos; DISCERN, *JAMA*, and GQS. All 3 scoring systems were positively correlated with each other, with moderate-strong correlations. The mean score was 1.63 (SD 0.56) for DISCERN, 1.30 (SD 0.56) for *JAMA*, and 1.71 (SD 0.84) for GQS. The mean scores are considered very low, indicating the poor quality of the videos. This finding has been replicated many times, with various studies finding TikTok videos of the health information being of poor quality. data shows that video length, daily views, and total views had a significant positive correlation with all 3 scoring systems.

This study also finds that 100% of the videos are created by personal accounts. This is alarming and indicates that either health care professionals are producing videos that are not going viral or being interacted with, or that health care professionals are not active on the topic. Health care professionals need to post more on the topic to spread proper awareness. Topical steroid phobia is an issue that has been increasing recently. A study published in *JAMA* indicates that its prevalence ranges between 21%-83%, indicating the fear that many patients may have against the use of steroids [29]. Although dermatologists remain the number one source of information according to a study, social media is contributing to a large part of our health information [30].

Some limitations of this study include assessing only the top 100 videos under the TSW hashtag on TikTok. To add, our study is designed more cross-sectionally and does not temporally follow certain accounts that regularly post about TSW. Several videos posted by the same account could have been studied to assess the progression of the disease, the fluctuation of the disease as well as the effectiveness of management options.

### Conclusions

Our study shows that although TSW TikTok videos have a huge reach, and a large potential to spread health information, the top 100 videos of TSW are of poor to very poor quality. The study shows as well that there exists no single definition for TSW, and the content on TikTok is vague and inconsistent, which is also reflected in the poor video quality. The duration of steroid use, symptoms experienced, and the management tools in most did not accord with the limited literature on TSW. The high reach of the videos about TSW, as well as their very poor quality may be a dangerous contributor to increased misinformation, particularly to the youths that have access to these videos, which may reflect poor health outcomes and disease management. Health care professionals are urged to be more active and post more videos on the topic on TikTok, either by having more accounts or commenting on the published ones, to spread scientific and evidence-based data.

## Abbreviations

GQS: Global Quality Scale
JAMA: Journal of the American Medical Association
TSW: topical steroid withdrawal

## Data Availability

The data sets generated during or analyzed during this study are available on request by emailing to the corresponding author.

## References

[1] Ventola CL (2014). Social media and health care professionals: benefits, risks, and best practices. P T.

[2] Zheng DX, Mulligan KM, Scott JF (2021). TikTok and dermatology: an opportunity for public health engagement. J Am Acad Dermatol.

[3] Nguyen M, Youssef R, Kwon A, Chen R, Park JH (2021). Dermatology on TikTok: Analysis of content and creators. Int J Womens Dermatol.

[4] Kanchan S, Gaidhane A (2023). Social media role and its impact on public health: a narrative review. Cureus.

[5] Hajar T, Leshem YA, Hanifin JM, Nedorost ST, Lio PA, Paller AS, Block J, Simpson EL (2015). A systematic review of topical corticosteroid withdrawal ("steroid addiction") in patients with atopic dermatitis and other dermatoses. J Am Acad Dermatol.

[6] Shrestha S, Joshi S, Bhandari S (2020). Prevalence of misuse of topical corticosteroid among dermatology outpatients. JNMA J Nepal Med Assoc.

[7] Karekar SR, Marathe PA, Nagarajan VB, Khopkar US, Chikhalkar SB, Desai PK, Dongre MS (2020). Use of topical steroids in dermatology: a questionnaire based study. Indian Dermatol Online J.

[8] Felner EI (2011). Reducing the risk for adrenal insufficiency in those treated for all: tapering glucocorticoids before abrupt discontinuation. J Pediatr Hematol Oncol.

[9] Spergel JM, Leung DY (2023). Topical steroid withdrawal syndrome: Should we worry?. Ann Allergy Asthma Immunol.

[10] Hwang J, Lio PA (2022). Topical corticosteroid withdrawal ('steroid addiction'): an update of a systematic review. J Dermatolog Treat.

[11] Nikookam Y, Guckian J (2021). TikTok™ and dermatology: lessons for medical education. Clin Exp Dermatol.

[12] Charnock D, Shepperd S, Needham G, Gann R (1999). DISCERN: an instrument for judging the quality of written consumer health information on treatment choices. J Epidemiol Community Health.

[13] Yaradılmış YU, Evren AT, Okkaoğlu MC, Öztürk Ö, Haberal B, Özdemir M (2020). Evaluation of quality and reliability of YouTube videos on spondylolisthesis. Interdiscip Neurosurg.

[14] Naseer S, Hasan S, Bhuiyan J, Prasad A (2022). Current public trends in the discussion of dry eyes: a cross-sectional analysis of popular content on TikTok. Cureus.

[15] Stacey SK, McEleney M (2021). Topical corticosteroids: choice and application. Am Fam Physician.

[16] Shakya Shrestha S, Bhandari M, Shrestha R, Thapa S, Karki A, Prajapati M, Shrestha S, Kc S, Karna D (2015). Study on corticosteroids use pattern in dermatological practice and investigating adverse effect of corticosteroids including its associated factors. Kathmandu Univ Med J (KUMJ).

[17] Walton RG, Farber EM (1961). Systemic use of corticosteroids in dermatology. Calif Med.

[18] Tracy A, Durrani S, Mir A (2022). Severe topical corticosteroid withdrawal syndrome or enigmatic drug eruption?. J Allergy Clin Immunol Pract.

[19] Sheary B (2018). Steroid withdrawal effects following long-term topical corticosteroid use. Dermatitis.

[20] Adcock IM, Brown CR, Barnes PJ (1996). Tumour necrosis factor alpha causes retention of activated glucocorticoid receptor within the cytoplasm of A549 cells. Biochem Biophys Res Commun.

[21] Dhar S, Seth J, Parikh D (2014). Systemic side-effects of topical corticosteroids. Indian J Dermatol.

[22] Coondoo A, Phiske M, Verma S, Lahiri K (2014). Side-effects of topical steroids: a long overdue revisit. Indian Dermatol Online J.

[23] Mehta AB, Nadkarni NJ, Patil SP, Godse KV, Gautam M, Agarwal S (2016). Topical corticosteroids in dermatology. Indian J Dermatol Venereol Leprol.

[24] Dey VK (2014). Misuse of topical corticosteroids: a clinical study of adverse effects. Indian Dermatol Online J.

[25] Rapaport MJ, Lebwohl M (2003). Corticosteroid addiction and withdrawal in the atopic: the red burning skin syndrome. Clin Dermatol.

[26] Eapen AA, Kloepfer KM, Leickly FE, Slaven JE, Vitalpur G (2019). Oral food challenge failures among foods restricted because of atopic dermatitis. Ann Allergy Asthma Immunol.

[27] Wang Z, Wang ZZ, Geliebter J, Tiwari R, Li XM (2021). Traditional Chinese medicine for food allergy and eczema. Ann Allergy Asthma Immunol.

[28] O'Connor C, Murphy M (2021). Scratching the surface: a review of online misinformation and conspiracy theories in atopic dermatitis. Clin Exp Dermatol.

[29] Li AW, Yin ES, Antaya RJ (2017). Topical corticosteroid phobia in atopic dermatitis: a systematic review. JAMA Dermatol.

[30] Choi E, Chandran NS, Tan C (2020). Corticosteroid phobia: a questionnaire study using TOPICOP score. Singapore Med J.

